# Gun-Shot Wound through the Bladder

**Published:** 1886-01-20

**Authors:** J. McF. Gaston

**Affiliations:** Professor of the Principles and Practice of Surgery in the Southern Medical College, Atlanta, Georgia


### Southern Medical Record.

EDITORS:
THOMAS S. POWELL, M. D. R. C. WORD, M. D.

*R. C. WORD, M.D., Managing Editor.*

☞All Communications and Letters on Business connected with the RECORD must be addressed to the Managing Editor.

VOL. XVI. ATLANTA, GA., JANUARY 20, 1886. No. 1.

### ORIGINAL AND SELECTED ARTICLES.

### GUN-SHOT WOUND THROUGH THE BLADDER.

BY J. MCF. GASTON, M. D.,
Professor of the Principles and Practice of Surgery in the Southern Medical College, Atlanta, Georgia.

I was called to Mr. A. B—— on the morning after he had been shot by a pistol in the left inguinal region, and learned that upon urinating shortly after receiving the injury a considerable discharge of blood had occurred, intermingled with the urine on this occasion and subsequently when he had passed water.

Upon inspecting the wound made by the ball, it was found that the orifice at which it entered was immediately above the transverse portion of the left os pubis, at a short distance within the line of the artery; and, upon palpation over the gluteal region of the opposite side, there was a point of sensitiveness indicating the site of the ball buried in the tissues. Thus it was evident that the ball had gone diagonally across the pelvis, traversing the walls of the bladder in its course; and, upon introducing a catheter through the urethra into the bladder, there was, at first, no flow of urine, whereupon it was withdrawn, and the opening was found clogged with clots of blood. On its being again passed into the bladder, blood mixed with urine flowed out, and thus it was verified that the tract of the ball had implicated the wall of the bladder in, perhaps, two points, by its entrance and exit. The bladder was most certainly greatly distended with urine when the shot was received, as the man had been visiting drinking saloons, with others, until the late hour at which the wound was inflicted, and thus it was raised up considerably above its normal position in the contracted state. The evacuation of the organ shortly after the shooting relieved it of this distention, and doubtless closed the orifices in its walls by the contractility of its tissues, so that there was no evidence of escape of urine through the external wound on the occasion of my examination next morning, and none of the effects of urinary infiltration into the cellular tissue followed the injury. After satisfying myself that no more blood or urine remained in the bladder, I directed that a flexible gum elastic catheter should be kept in the urethra, so as to keep the urine drawn off, and thus allow of the consolidation of the orifices in the walls of the bladder by its continued contraction. The external opening was treated simply with lint moistened with a 2-per cent. solution of carbolic acid, and, when febrile reaction ensued, 5 drops of tincture of aconite were taken every two hours. On the third day, the bowels not having acted, he took a tablespoonful of Epsom salts, and the case progressed without any untoward symptoms. After the lapse of a few days, the catheter was dispensed with, and he was directed to pass his urine regularly every three hours, so as to prevent accumulation.

The progress of the case was entirely satisfactory, in the prompt healing of the external wound, without suppuration ; and, at the end of a month the patient was up and able to attend to business. I have met him on the street within a few days—now three months since the injury—and he states that no inconvenience whatever is experienced, and that he is entirely well in all respects.

